# Participant recruitment among vulnerable populations: An experience from a qualitative study among refugee adolescents in Germany

**DOI:** 10.12688/openreseurope.14654.2

**Published:** 2023-11-07

**Authors:** Marjan Mohammadzadeh, Asra Taras, Christiane Stock

**Affiliations:** 1 Institute of Health and Nursing Science, Charité – Universitätsmedizin Berlin, corporate member of Freie Universität Berlin and Humboldt-Universität zu Berlin, Berlin, 13353, Germany; 2 Unit for Health Promotion Research, University of Southern Denmark, Dengevej 14, 6705, Esbjerg, Denmark

**Keywords:** Hard to reach populations, Qualitative study, Participant recruitment, Refugee adolescents, Vulnerable populations.

## Abstract

When it comes to actual practice, comparing to theoretical plans, the study progress is much more complicated due to both predicted and unpredicted challenges in the study participant recruitment. The situation is even more difficult if the research team deal with a vulnerable population. The current article represents and discusses the recruitment process for a currently unpublished qualitative study, conducted in Germany in 2021, as the first phase of our project: SC-SD4ASA: A self-care/self-development guidebook for asylum-seeking adolescents. First a brief of the study methodology is outlined and then, the strategies used to reach the potential participants including major/government organizations for refugees, private organizations and local centers, social media, youth’s favorite public places, and participatory strategy are discussed. Finally, our experience of participant recruitment challenges in this study is reviewed. We hope that the points raised from our experience can be helpful to other researchers in the same field. Overall, the experience of recruiting our participants emphasized the necessity for a dedicated point of contact for consultation and the provision of resources to connect with potential participants for researchers in the field of refugee and migrant health. Furthermore, the influence of trustworthy individuals, particularly within the community, should not be underestimated. Their assistance can significantly benefit the advancement of a research project.

## Introduction

Study participants are the crucial inseparable part of studies related to human nature. Recruiting participants, including planning, targeting, and enlistment to employ the volunteers accounts for a significant part of the data collection. This is usually one of the most challenging stages of any longitudinal research study due to the limitations of the time and effort in research studies ( Patel *et al.,* 2003a). An appropriate recruitment strategy can lead to an on time and efficient data collection and also avoid losing a considerable number of participants in a long duration of the process. Furthermore, having such strategies decreases ethical challenges and improves the research interpretation and validity of the results ( Bonk, 2010).

Overall, study approach and method, study population, sample size, characteristics and preferences of participants, and recruitment strategies are some of the critical factors defining the success of including volunteer individuals in a study ( Patel *et al.,* 2003b). Based on these factors, a broad-spectrum of recruitment strategies can be utilized to assist participant engagement and enrolment in research.

Study population or target population is defined as “the total group of individuals from which the sample might be drawn” ( Tipape & Kepha, 2016). It can be explained based on the study location, gender, age, educational level, religion, specific health condition, or any other determining characteristic(s) that could logically separate them from other groups in a community ( Zhao *et al.,* 2013). To put it simply, individuals in a study population typically share certain characteristics or factors related to the study's goals. However, scientific studies usually focus on a 'sample' of individuals from the larger population rather than everyone. Various sampling methods are available, depending on the study's approach and goals ( Majid, 2018).

However, in almost all research, when it comes to ‘actual practice’, compared with theoretical plans and proposal writing, more and less the study progress is much more difficult due to predicted or unpredicted challenges in the study sample recruitment ( Sarstedt *et al.*, 2018). The situation is even much more challenging when the research team deal with a hard-to-reach vulnerable population.

There are some terms used by different researchers to name ‘not-easy-to-reach’ populations and groups, such as hard-to-reach, vulnerable, disadvantaged, underrepresented, defenseless, exposed, marginalized, or at-risk groups ( Nguyen Thanh *et al.,* 2019). But whatever we call them, from the health and social studies’ point of view, they all have a feature in common: being at higher risk of unsatisfactory physical and psychological health as well as of poor social status compared to other groups ( Ellard-Gray *et al.,* 2015). Based on this definition, several community groups can be potentially placed in this category. For example, patients with incurable diseases, disabled people, the homeless individuals, asylum seekers, and refugees.

The vulnerable populations also have another shared characteristic when it comes to health and social studies. Based on the reports of previous studies, these groups of community are much more difficult to find, convince, and recruit as research subjects. Several researchers believed that, based on their experiences, field research among vulnerable populations is more problematic and challenging due to various reasons such as the requirement of special ethical considerations, high probability of denied access, refusal, or incomplete collaboration ( Fête *et al.,* 2019).

This article presents and discusses the recruitment process for the qualitative study, as the first phase of our three-phase project: ‘SC-SD4ASA: A self-care/self-development guidebook for asylum-seeking adolescents’ conducted in Germany in 2020–2022. The protocol of all three phases is represented in the 56th annual meeting of the German Society for Social Medicine and Prevention (DGSMP) as a poster and published in 2021 ( Mohammadzadeh *et al.,* 2021). At the moment, the collected data of the first phase is analyzed, and the results will be published soon.

As the essay outline, first the study sampling and methodology is briefly explained and then, the strategies used to reach the potential participants are discussed. Finally, our experience of participant recruitment challenges in this study is reviewed. 

## A brief of the SC-SD4ASA study

### Ethical approval

The project’s ethics approval was obtained from the research ethical committee of Charité - Universitätsmedizin Berlin (number EA2/087/21) on 08.06.2021. Participants and their parents/guardians received full details of the project process in both oral (face to face meetings) and written forms.

The study was conducted from August to December 2021 in Germany. The main objective of this study was to assess self-care and self-development knowledge, experience, and needs among asylum seeking adolescents in Germany through three different perspectives: phenomenology, cultural, and gender perspectives ( Mohammadzadeh *et al.,* 2022). To achieve this goal, an intra-triangulation approach including three independent qualitative studies of in-depth interviews, focus group discussions, and photovoice methods was used. Following the purposive homogeneous sampling method, the study participants were eligible asylum-seekers aged 15 to 19 years old, who volunteered to participate in the project.

All the project documents including information sheets, consent forms, and posters were prepared in four different languages: Deutsch, Arabic, Farsi, and English. The interview and group discussions were planned to be held in these four languages as well; however, almost all the data collection were done in Farsi (for the Iranian and Afghan participants) and Arabic (for the participants form Arabic countries including Syria, Palestine, and Lebanon). Only in one case did the participant in the photovoice data collection ask for the English documents.

Data was collected using the following approaches and methods:

1.    Qualitative data were collected from interviews (21 participants aged 14 to 19 from five countries) and focus group discussions (four groups with 26 participants in total). The interviews were conducted in both online and face to face form, in the average time of 48 minutes. The average time of the group discussions was two hours and 45 minutes, and all four sessions were conducted face to face.

2.    Visual data (photovoice approach) were collected according to the SHOWeD method ( Liebenberg, 2018). We received 41 photos from 15 participants (each participant sent one to five photos).

To obtain more comprehensive data for this study, each participant was placed in only one of the interview or group discussion groups. However, due to the different nature of photography data, the participants in this group could also be involved in interviews or group discussions. Except one, all the participants in the photovoice study previously participated in either an interview or group discussions ( Mohammadzadeh *et al.,* 2023).

## Experiences with participant recruitment resources and strategies

There were five main source groups contacted in the initial study in order to search for potential participants.

### 1. The major/government organizations which are directly or indirectly related to refugee adolescents and/or refugee families

Searching for the potential participants, our first group of resources were the major organizations which are, directly or indirectly, related to refugees’ families and youth. In order to reach out to these organizations, a comprehensive online search in English and German was done by the research team, resulting in a list of the names, addresses, emails, and phone numbers of the organizations. In the first step, we tried to contact all these organizations, directly by phone. However, except in two cases, after several attempts, nobody picked up the phone. In two cases where we could have a phone conversation with the person in charge, the respondents asked us to send our request through an official email. And, of course, due to the special circumstances of the epidemic, we were not allowed to meet them in person in any case.

In the next step, an official collaboration request email (in German), was sent to all email addresses, along with the ethical approval letter, a detailed project information sheet, and consent forms in four different languages (Deutsch, English, Arabic and Farsi). However, even after follow-up emails/phone calls, we received no reply, neither positive nor negative, from any of these organizations regarding our request. Obviously, these major organizations, for any yet to be explored reason, were reluctant to get involved in research projects.

### 2. Private organizations, non-governmental organizations (NGOs), refugee residentials, and local youth centers

Our next source to find our potential participants were private organizations, non-governmental organizations (NGOs), refugee residentials, and local youth centers. As the host institute was in Berlin, we mostly (but not only) focused on Berlin and Brandenburg. As most of the local youth centers did not have enough information to be found through an internet search, we also posted some announcements *via* social media (such as Facebook ^1^) and instant messaging Apps (such as Telegram and WhatsApp groups) in German, Farsi, and Arabic looking for the staff of these centers. We also contacted the religious and cultural centers in this area which are usually the gathering place of refugee youth or their families. The feedback we received from these centers was different and, in some cases, considerable. While the project was expected to be welcomed by the centers due to its educational and health promoting nature, many of them expressed their unwillingness to cooperate under various pretexts. In some cases, they cited the weaknesses and problems of refugee adolescents as the reason for their unwillingness to cooperate. They mentioned reasons like ‘these teens usually do not tell the truth’, or that ‘these issues are too hard for them to understand’. This kind of feedback raised our concerns because we consider these statements as stereotyping refugee adolescents, and therewith as unethical and unprofessional, especially when expressed by those who are in direct contact with the refugee community. Moreover, in some cases, these centers asked us for several documents such as the full interview protocol and, after receiving that, just ignored all our follow-ups.

The situation remained relatively consistent when contacting youth centers, NGOs, refugee residences, and private organizations through email to request their cooperation. From about 20 emails to different addresses, we received only a few replies, mentioning that they don’t have access to refugee adolescents or, in the best case, referring us to institutes and organizations we had already contacted. Our attempt to obtain permissions to enter the accommodation centers and speak directly to families was unsuccessful for various reasons, including pandemic restrictions.

### 3. Social media announcements

Simultaneous with the above two strategies, as a direct participant recruitment method, an announcement of volunteer recruitment was posted on several social networks common among immigrants and refugees along with the project informative poster. The initial reception of these announcements was relatively satisfactory. Candidates received project information sheets in one of the four languages, English, German, Persian, or Arabic, *via* private message, and their possible questions were answered in their desired language. However, despite the initial response, the final result was not very successful and less than 25% of the total participants were attracted through this method. The main reason for the relative failure, especially for volunteers outside Berlin, seemed to be the difficulties related to the study consent forms. Candidates were asked to send a postal address to the research team to receive the consent forms. For obvious reasons, families could hardly trust to send their address and names to a stranger, even though they knew we were working for a well-known organization.

And even when they had trust in the researchers, most teens or their families had no incentive to spend time posting signed forms (although a stamped empty envelope with our address was sent to the candidates along with the consent forms and they only needed to drop the envelope containing the signed forms in a mailbox). Despite these restrictions, about a quarter of the participants were recruited for the project through this strategy.

### 4. Participatory data collection strategy

In our experience, this strategy was a game changer, resulting in about 70 % of the study participants. Since the trust factor seemed to be one of the main pillars of volunteer recruitment among refugee families, this time, we tried to use the snowball method. In this method we went forward through people who, on one hand, knew one of the research team members and on the other hand, had a relationship with refugee families to gain the participants' trust. As a result, a few families were introduced to us who, due to their trust in the person who introduced them, were not worried about their children's participation in this project.

This process helped us to meet some refugee adolescents who, in addition to participating in the study, volunteered to assist the research team in recruiting other participants. Having the experience of participating in the interview, they introduced the project to their friends and invited them to participate in the interviews or group discussions. This process led to a chain of teens who trusted each other and wanted to share their friends' experiences. Moreover, the participants' familiarity and trust in each other had another advantage. The group discussion sessions took place in an environment full of intimacy and trust, which led to the collection of valuable and rich information. At the suggestion of the teenagers themselves, a telegram group was created for them to be able to communicate more with each other. This telegram group in turn became an incentive to attract more volunteers.

### 5. Youths favorite public places

Although the participant recruitment process significantly progressed following the participatory data collection strategy, data saturation was not achieved in one of the language groups. To complete the process in this group, on four weekends the project was introduced to the eligible adolescents in several public youth gathering centers (shopping malls, for example). In this method, the voluntary cooperation of two adolescents who had previously participated in the interview was used. However, recruiting participants in this way was very time consuming and slow. On average, one in 10 adolescents was willing to listening project-related information, and two out of five expressed their willingness to participate after hearing the information. Yet, the number of people who agreed to sign the consent form and eventually participate in the interview or group discussion was even less. However, after four weeks, data saturation was achieved in the desired group. Thus, the process of data collection was successfully completed after more than four months.

## Discussion

Not all researchers believe that participant recruitment and data collection among vulnerable groups is more challenging compared with the non-vulnerable papulations ( Chamberlain & Hodgetts, 2018), but several field experiences, including our study ( Mohammadzadeh *et al.,* 2021), show otherwise ( Cheema *et al.,* 2018; Fête *et al.,* 2019; Nguyen Thanh *et al.,* 2019; Mohammadzadeh *et al.,* 2020).

Our experience has shown that most centers dealing with refugee families and children are reluctant to collaborate or engage in community projects, even as much as just introducing the project or distributing project posters. This issue alone could be the subject of future research projects; however, we believe that the high workload and the multiplicity of tasks and clients make employees reluctant to engage in new tasks that are not clearly defined as their duties and are considered overtime in practice. In the case of our project, the specific situation due to the limitations and problems of the pandemic period exacerbated the issue.

Generally, regarding the effective relationship as well as negotiating and gaining access to hard-to-reach quantitative data, between the research team and the organizations which are considered as the “gatekeepers” the concept of “goodwill” was highlighted by Crowhurst & Kennedy-Macfoy, 2013, especially when a researcher seeks to gain access to secondary quantitative data. Goodwill in this context depends on both the researcher's emotional reactions to the gatekeepers and the support provided by the gatekeepers in facilitating the search for data ( Crowhurst & Kennedy-Macfoy, 2013). Researchers might find it valuable to consider this concept or approach when circumstances allow However, due to the intricate and ever-changing nature of interactions in the field, it can be asserted that there is no one-size-fits-all approach to accessing gatekeepers. Some studies recommend that reflexivity, centered on the social identities of researchers, gatekeepers, and participants, is valuable in understanding the intricate interactions, potential conflicts, and cooperation involved in the gatekeeping process ( Turhan & Bernard, 2020).

In the case of smaller and local centers, it seems that they prefer not to engage in such activities to avoid any possible problems and troubles. However, the fact that these centers in some cases, based on their own judgment, did not consider refugee adolescents suitable for this project, or even labeled them with negative characteristics, such as not telling the truth, to justify their non-cooperation, needs special consideration.

However, a part of our unsuccessful experience regarding the centers was due to the study limitations. Undoubtedly, our list of public and private centers related to adolescents was not complete and we did not have the information of many of these centers. It is also possible that our emails were not sent to the correct address or department or that our contact information was not updated correctly.

The relatively unsuccessful experience in our recruitment strategy using social media announcements was to some extent expected by the research team. Many refugee families, for a variety of reasons, prefer to avoid interviews as much as possible, which may lead to the disclosure of some of their personal information and, of course, try to keep their children away from such situations, in which they could speak about personal issues. Despite our emphasis that the interviews’ and group discussions’ results have no positive or negative effects on their asylum-seeking process, following the saying that, ‘if it ain’t broke, don’t fix it’, these families preferred not to take any risk in the middle of their uncertain situation. For these families, the concept of an ‘interview’ was much different from our concept as researchers. For them, the concept of the interview was reminiscent of the anxious moments that they had in the asylum-seeking process with different authorities such as immigration officers, border police, social workers, and many others. It, therefore, seemed understandable that they were reluctant to take the risk of a similar experience. This barrier was even more pronounced when the families figured out that they and their adolescents need to sign formal consent forms.

When it comes to the adolescents, we faced other challenges. For the adolescents, especially the younger ones, being volunteered is all about seeing some changes or benefits. As adolescents mostly live in the present ( Moen & Hall-Lord, 2019), it is difficult to persuade them to take part in an activity that has no immediate benefit or visible change for them ( Eisenstein, 2018), as we emphasized in our information sheet. Analyzing the importance of a scientific research, especially on an unfamiliar subject, through tedious explanation of the project information sheet may not be easy for most of them, even when it is written in an easy language. Of course, the situation for refugee adolescents is even more complicated due to their avoidance of any possible risk.

On the other hand, after the interview or group discussion sessions, our participants mostly stated that the topic of discussion was more interesting and useful than they thought. For all these adolescents, it was the first time to experience participation in a research interview or discussion. Clearly, in the duration of the interviews or group discussions they had gotten a better view about the importance of their action as volunteers. 

 So, we realized that we need something more realistic and understandable than saying that ‘there is no immediate benefit for the participants in this study. The results of this study could be useful to you and your peers in the future’. to encourage them to engage in an interview. Giving ‘a gift’, such as a small shopping voucher, mostly was considered as a common way to say ‘thank you for your participation and time’, in many research studies ( Schondel & Boehm, 2000). However, these gifts seemed to be an important motivator for the participants, especially younger ones. But the success of the participatory data collection strategy showed that the approval of a trusted person or friend was the strongest incentive to participate in the study. As one of our participants said ‘I don’t need extra explanation about the project. My best friend told me the interview was fun and interesting’. Other research support our experiences that building trust is essential in engaging vulnerable population groups in participatory research projects ( Christopher *et al*., 2008; Rivas-Drake *et al.*, 2016).

So, an appropriate motivation/encouraging factor could help the researchers to grasp the potential participants’ attention and buy some time for more explanation about the project. But it should be selected wisely and with full consideration of ethical parameters, so that the encouragements do not play the role of bribes. Moreover, an appropriate encouragement may be very different from one target population to another.

Overall, our participants’ recruitment experience among refugee adolescents in Germany highlighted the need for a specific reference for consultation, as well as the introduction of sources to access to potential participants for the researchers in the field of refugees’ and migrants’ health, who make up a significant part of Germany's population. In addition, these authorities can be reliable intermediaries for introducing approved projects to refugee families. In this regard, having a database of reference centers could be highly useful.

Moreover, in non-clinical research projects among healthy children and adolescents, especially in the vulnerable groups, replacing the traditional methods, such as information sheets, which are mostly long and confusing, with innovative and easy to understand methods to introduce the project and its importance, such as using short videos, color images and infographics, might be beneficial. Our experience has shown that adolescents are very active and interested when they understand the significance of their participation and value of their thoughts. However, understanding this importance through formal explanations is not always easy. 

And finally, the power of trustworthy people, especially within the community, should not be overlooked. The help of these people can be a great advantage to the progress of the project. In the case of children and adolescents, using the help of interested friends and peers can even be more effective and even be considered as an efficient motivator.

### Ethical considerations

While the use of these recommendations may be effective in improving the efficiency of participant recruitment methods, the priority of ethical considerations should be carefully considered. There is no doubt that this matter is much more sensitive in vulnerable groups. Although individuals and organizations involved with vulnerable groups are expected to be familiar with ethics and personal data protection rules, providing the information about the specific ethical considerations of each project to individuals outside the research team is essential.

This information should be provided to volunteers, as needed, in the form of the project sheet or during briefing meetings or training sessions. The protection of personal data and information must always precede any action taken in the data collection. While introducing people eligible to participate in the study or alternatively, introducing the study to them, can be done in a variety of ways, the personal identification of the participants must be carefully protected and secured by the research team. Ultimately, any dissemination of the study findings must align with the informed consent agreements established between the participants and the research team.

### Study limitations

In addition to the limitations mentioned in the previous sections of this article, the recruitment of participants in this study also faced other limitations. Schools are one of the most important and reliable sources for employing participants, especially in the age group of children and adolescents. However, due to the coincidence of this study with the closure of schools due to epidemic conditions, it was not possible to use the capacity of schools to hire participants. Of course, this restriction also included other educational and cultural centers related to refugee adolescents and their families. Moreover, due to the pandemic travel restrictions, direct access to potential participants and organizations outside of Berlin and nearby cities was difficult.

## Conclusion

The results obtained from conducting scientific research among vulnerable groups can be a valuable foundation for the success of the plans aiming to address the specific problems of these groups. However, studies among vulnerable groups generally face many challenges at various stages, including recruiting participants. Enhancing recruitment methods to establish greater trust within the study population can lead to successful retention in endeavors. Effectively conducting research with the participants' best interests in mind may lead to the creation of a pool of individuals who will willingly participate in studies with a similar target group. As retention rates improve, word-of-mouth recommendations, particularly within vulnerable populations, can become a valuable recruitment tool ( Andrews & Davies, 2022). We hope that the points raised from our experience can be helpful to other researchers in the same field. It should be noted that the use of a combination of methods introduced in this study in addition to other appropriate methods can effectively accelerate the process.

## Ethical approval

 The project’s ethics approval was obtained from the research ethical committee of Charité – Universitätsmedizin Berlin (number EA2/087/21) on 08.06.2021. Participants and their parents /guardians receive full details of the project process in both oral (face to face meetings) and written forms.

## Data Availability

No data are associated with this article.
