# Supplementary material for: Participant recruitment among vulnerable populations: An experience from a qualitative study among refugee adolescents in Germany
Source: Open Res Eur. 2023 Nov 7;2:47. Originally published 2022 Apr 8. [Version 2] doi: 10.12688/openreseurope.14654.2 (PMC10652035; doi:10.12688/openreseurope.14654.2)
Supplement: Peer review history

# Reviewer report: Reviewer response for version 2

**DOI:** 10.21956/openreseurope.18138.r36120

**Published:** 2023-11-28

**Authors:** Todd H Davies

**Affiliations:** 1 Department of Family and Community Health, Joan C Edwards School of Medicine, Marshall University, Huntington, WV, USA

Author revisions are responsive.

Is the argument persuasive and supported by appropriate evidence?

Partly

Is the work clearly and cogently presented?

Partly

Is the topic of the essay discussed accurately in the context of the current literature?

Partly

Does the essay contribute to the cultural, historical, social understanding of the field?

Yes

Reviewer Expertise:

Clinical research in vulnerable populations

I confirm that I have read this submission and believe that I have an appropriate level of expertise to confirm that it is of an acceptable scientific standard.

# Reviewer report: Reviewer response for version 2

**DOI:** 10.21956/openreseurope.18138.r36118

**Published:** 2023-11-15

**Authors:** Pilar Serrano-Gallardo

**Affiliations:** 1 Nursing Department, Faculty of Medicine, Universidad Autonoma de Madrid, Madrid, Spain

In response to the authors' comments, I think the word "Obviously" should be removed from the sentence: “Obviously, these major organizations, for any yet to be explored reason, were reluctant to get involved in research projects” because this seems to come from preconceived ideas. Furthermore, as the authors themselves point out, they have not explored these reasons.

 Although the authors can point out their impressions in light of their experiences, given that this work did not aim to explore the stereotypes of refugee adolescents in different organizations and centers, it would be advisable to modify the wording of the sentence: “This kind of feedback raised our concerns because we consider these statements as stereotyping refugee adolescents, and therewith as unethical and unprofessional, especially when expressed by those who are in direct contact with the refugee community”. As it appears now, it leads one to think that these statements could be common.

Is the argument persuasive and supported by appropriate evidence?

Partly

Is the work clearly and cogently presented?

Partly

Is the topic of the essay discussed accurately in the context of the current literature?

Partly

Does the essay contribute to the cultural, historical, social understanding of the field?

Yes

Reviewer Expertise:

Public Health research in vulnerable population

I confirm that I have read this submission and believe that I have an appropriate level of expertise to confirm that it is of an acceptable scientific standard.

# Reviewer report: Reviewer response for version 2

**DOI:** 10.21956/openreseurope.18138.r36119

**Published:** 2023-11-15

**Authors:** Maura Dowling

**Affiliations:** 1 School of Nursing and Midwifery, National University of Ireland, Galway, Galway, Ireland

I have reviewed the new version, and the status can be updated to Approved.

Is the argument persuasive and supported by appropriate evidence?

Yes

Is the work clearly and cogently presented?

Yes

Is the topic of the essay discussed accurately in the context of the current literature?

Yes

Does the essay contribute to the cultural, historical, social understanding of the field?

Yes

Reviewer Expertise:

NA

I confirm that I have read this submission and believe that I have an appropriate level of expertise to confirm that it is of an acceptable scientific standard.

# Reviewer report: Reviewer response for version 1

**DOI:** 10.21956/openreseurope.15829.r30404

**Published:** 2022-12-23

**Authors:** Pilar Serrano-Gallardo

**Affiliations:** 1 Nursing Department, Faculty of Medicine, Universidad Autonoma de Madrid, Madrid, Spain

This work raises an important issue for research with vulnerable people, where it is difficult to recruit participants. The scientific community needs key elements to work in this field, and this essay provides important findings.

 In a "Responsible Research approach" it is essential to incorporate participatory processes throughout all stages of research, and this is especially relevant with vulnerable populations. Considering what is reflected in the section “Experiences with participant recruitment resources and strategies”, it does not seem that the different source groups have been involved in the investigation from the outset, being part of the design of the recruitment strategies themselves. It does not seem appropriate for the authors to claim that it was obvious that some strategies would not work, because if they did, they should not have been incorporated. These issues should be part of Discussion, as they may be part of the reasons for the poor success of several of the strategies. Nor does it seem prudent to make judgments such as “This kind of feedback raised our concerns because we consider these statements as stereotyping refugee adolescents, and therewith as unethical and unprofessional, especially when expressed from those who are in direct contact with the refugee community”, given that no research has been carried out on these issues.

 On the other hand, the authors should also provide more details on what the "Participatory Data Collection Strategy" consisted of, since it seems to have produced the best results.

 Some further reading is recommended, which may be particularly relevant in the context of this essay:

- National Institute for Health Research. About INVOLVE—INVOLVE [Internet]. Available online: https://www.invo.org.uk/about-involve/
- Jagosh, J.; Macaulay, A.C.; Pluye, P.; Salsberg, J.; Bush, P.; Henderson, J.; Sirett, E.; Wong, G.; Cargo, M.; Herbert, C.P.; *et al.* Uncovering the benefits of participatory research: Implications of a realist review for health research and practice. Milbank Q. 2012, 90, 311–346.
- Kramer, C.B.; LeRoy, L.; Donahue, S.; Apter, A.J.; Bryant-Stephens, T.; Elder, J.P.; Hamilton, W.J.; Krishnan, J.A.; Shelef, D.Q.; Stout, J.W.; *et al.* Enrolling African-American and Latino patients with asthma in comparative effectiveness research: Lessons learned from 8 patient-centered studies. J. Allergy Clin. Immunol. 2016, 138, 1600–1607.
- Serrano-Gallardo, P.; Cassetti, V.; Boone, A.L.D.; Pisano-González, M.M. Recruiting Participants in Vulnerable Situations: A Qualitative Evaluation of the Recruitment Process in the EFFICHRONIC Study. Int. J. Environ. Res. Public Health 2022, 19, 10765. https://doi.org/10.3390/ijerph191710765

Regarding other minor issues, abstract should incorporate the main conclusions related to the recruitment process analyzed. Citation is introduced in the text ((Mohammadzadeh *et al.*, unpublished) which does not make much sense given that it is not yet accessible as it is unpublished work.

Is the argument persuasive and supported by appropriate evidence?

Partly

Is the work clearly and cogently presented?

Partly

Is the topic of the essay discussed accurately in the context of the current literature?

Partly

Does the essay contribute to the cultural, historical, social understanding of the field?

Yes

Reviewer Expertise:

Public Health research in vulnerable population

I confirm that I have read this submission and believe that I have an appropriate level of expertise to confirm that it is of an acceptable scientific standard, however I have significant reservations, as outlined above.

## References

[1] : Uncovering the benefits of participatory research: implications of a realist review for health research and practice. *Milbank Q* .2012;90(2) : 10.1111/j.1468-0009.2012.00665.x 311-46 10.1111/j.1468-0009.2012.00665.x 22709390PMC3460206

[2] : Enrolling African-American and Latino patients with asthma in comparative effectiveness research: Lessons learned from 8 patient-centered studies. *J Allergy Clin Immunol* .2016;138(6) : 10.1016/j.jaci.2016.10.011 1600-1607 10.1016/j.jaci.2016.10.011 27789250

[3] : Recruiting Participants in Vulnerable Situations: A Qualitative Evaluation of the Recruitment Process in the EFFICHRONIC Study. *Int J Environ Res Public Health* .2022;19(17) : 10.3390/ijerph191710765 10.3390/ijerph191710765 36078487PMC9518307

# Response

**Published:** 2023-10-24

**Authors:** Marjan Mohammadzadeh

- **In a "Responsible Research approach" it is essential to incorporate participatory processes throughout all stages of research, and this is especially relevant with vulnerable populations. Considering what is reflected in the section “Experiences with participant recruitment resources and strategies”, it does not seem that the different source groups have been involved in the investigation from the outset, being part of the design of the recruitment strategies themselves. It does not seem appropriate for the authors to claim that it was obvious that some strategies would not work, because if they did, they should not have been incorporated. These issues should be part of Discussion, as they may be part of the reasons for the poor success of several of the strategies. Nor does it seem prudent to make judgments such as “This kind of feedback raised our concerns because we consider these statements as stereotyping refugee adolescents, and therewith as unethical and unprofessional, especially when expressed from those who are in direct contact with the refugee community”, given that no research has been carried out on these issues.**

  Not all the data collection strategies used in this study were considered "participatory." The participatory approach was just one part of our data collection strategy, but it turned out to be very effective. We didn't initially plan to involve as many different sources as we eventually did. Given the vulnerability of the population, adding more sources could increase the risk, especially considering we already used over five different sources for data collection. We weren't sure about the effectiveness of our methods until we tried them. The observations and experiences we shared in this paper, such as noting that "major organizations were reluctant to get involved in research projects," were the results of our own experiences and observations, not preconceived ideas or prior knowledge. The feedback we received regarding some refugee centers was based on our real experiences and interactions. Other researchers may have different experiences and perspectives on these matters. In conclusion, we view this article as a reflection of our honest experiences during the data collection process rather than a theoretical discussion of data collection methods. Our hope is that this article can assist researchers in the same field in navigating the process more efficiently.  

- **Regarding other minor issues, abstract should incorporate the main conclusions related to the recruitment process analyzed. Citation is introduced in the text ((Mohammadzadeh et al., unpublished) which does not make much sense given that it is not yet accessible as it is unpublished work.**

The abstract is modified. We also replaced the citation as it is published now.

# Reviewer report: Reviewer response for version 1

**DOI:** 10.21956/openreseurope.15829.r30401

**Published:** 2022-11-29

**Authors:** Todd H Davies

**Affiliations:** 1 Department of Family and Community Health, Joan C Edwards School of Medicine, Marshall University, Huntington, WV, USA

The authors provide an important example of how standard methods for recruiting participants into clinical research can be ineffective in vulnerable populations.

 The key finding was that trust was a critical factor in recruiting their target population, whom they labeled as vulnerable. However, the authors do not explore an extensive trust literature with vulnerable populations (citations added). Nor do they fully explain the vulnerable nature of the adolescent population. "The power of trustworthy people" in research is an important discussion point that should be explored more in the context of future clinical research approaches.

 There are several ethical considerations involving coercion of this population: 1. Utilizing peer pressure and 2. The value of gift cards to this population. These are reasonable approaches, but considering this manuscript is specifically about vulnerable populations, some explanation of these how these things were managed is appropriate.

Is the argument persuasive and supported by appropriate evidence?

Partly

Is the work clearly and cogently presented?

Partly

Is the topic of the essay discussed accurately in the context of the current literature?

Partly

Does the essay contribute to the cultural, historical, social understanding of the field?

Yes

Reviewer Expertise:

Clinical research in vulnerable populations

I confirm that I have read this submission and believe that I have an appropriate level of expertise to confirm that it is of an acceptable scientific standard, however I have significant reservations, as outlined above.

## References

[1] : Rigor and Respect: Recruitment Strategies for Engaging Vulnerable Populations in Research. *J Interpers Violence* .2022;37(17-18) : 10.1177/08862605211023497 NP17052-NP17072 10.1177/08862605211023497 34107811

[2] : Factors Influencing Participation in Clinical Research: Do minority and lower socioeconomic status patients experience greater barriers in participation. *Poster presented at the LVHN Research Scholar Program Poster Session, Lehigh Valley Health Network, Allentown, Pennsylvania* .2014; Reference source

[3] : "A scarlet letter": Stigma and other factors affecting trust in the health care system for women seeking substance abuse treatment in a community setting. *Subst Abus* .2019;40(2) : 10.1080/08897077.2018.1544184 170-177 10.1080/08897077.2018.1544184 30759047

[4] : Race, socioeconomic status, and health: complexities, ongoing challenges, and research opportunities. *Ann N Y Acad Sci* .2010;1186: 10.1111/j.1749-6632.2009.05339.x 69-101 10.1111/j.1749-6632.2009.05339.x 20201869PMC3442603

[5] : Participant recruitment and retention from vulnerable populations in clinical trials is a matter of trust. *Contemp Clin Trials* .2022;123: 10.1016/j.cct.2022.106969 106969 10.1016/j.cct.2022.106969 36273802

# Response

**Published:** 2023-10-24

**Authors:** Marjan Mohammadzadeh

- **The key finding was that trust was a critical factor in recruiting their target population, whom they labeled as vulnerable. However, the authors do not explore an extensive trust literature with vulnerable populations (citations added). Nor do they fully explain the vulnerable nature of the adolescent population. "The power of trustworthy people" in research is an important discussion point that should be explored more in the context of future clinical research approaches.**

Additional points and highlights related to the requested modifications have been included in the conclusion section and we have included additional literature addressing trust.

- **There are several ethical considerations involving coercion of this population: 1. Utilizing peer pressure and 2. The value of gift cards to this population. These are reasonable approaches, but considering this manuscript is specifically about vulnerable populations, some explanation of these how these things were managed is appropriate.**

  It's worth noting that throughout our process, we did not encounter any instances of peer "pressure," Peer pressure would be significantly distant from the goals of our project. Rather, what we observed was peer support and trust, as you astutely pointed out in your previous comment. Our participants appeared happier and more at ease working together, as explicitly discussed in the paper under "Experiences with participant recruitment resources and strategies," part 4, paragraph 2. The” value of gift cards” is based on similar studies conducted in Germany and endorsed by the ethical committee. Nonetheless, various considerations regarding this matter are discussed in paragraph 10 of the discussion section.

# Reviewer report: Reviewer response for version 1

**DOI:** 10.21956/openreseurope.15829.r29021

**Published:** 2022-04-25

**Authors:** Maura Dowling

**Affiliations:** 1 School of Nursing and Midwifery, National University of Ireland, Galway, Galway, Ireland

The discussion paper by Mohammadzadeh and colleagues presents a very interesting overview of the challenges in recruiting vulnerable populations for a qualitative study in a pandemic. The various strategies employed by the research team to recruit refugee adolescents in Germany for their study are discussed and obstacles are highlighted. In addition, the challenges to recruitment in a time of restrictions during the COVID-19 pandemic are presented.

 I have some minor comments for consideration that will further strengthen this paper.

- The authors should reframe the discussion on the challenges of the first two approaches (major government and private organisations) in the context of gatekeepers. The discussion could be strengthened by reference to insights provided by Crowhurst and Kenney-MacFoy (2013) and the findings of Snowden and Young (2017) on gatekeeping as a continuum from unconscious to conscious.
- Some attention to research reflexivity would also strengthen the discussion on accessing participants via gatekeepers and any possible power dynamics. The paper by Turhan and Bernard (2020) offers guidance on approaching reflexive accounts.

- The participatory data collection strategy was found to be the most successful recruitment strategy. It would be interesting to mention any details of including some refugee adolescents on the research team when designing the study (public and patient involvement [PP1]). You may have done this or it may not have been possible due to the pandemic, but it is not clear from the account provided.

- Rephrasing of the following sentences is needed where the meaning is not entirely clear: a) The last sentence in the section ‘ethical considerations’; ‘ …according to the informed consent forms…’ ; b) Discussion, paragraph 5, the first sentence; what does ‘more and less predictable’ mean?
- Some minor typos noted as follows: a) Introduction, paragraph 3, sentence beginning with ‘In other words…in one or more characteristic [sic]’; b) Introduction, paragraph 3, sentence beginning with ‘However, scientific studies …instead of the whole populations [sic]’

Is the argument persuasive and supported by appropriate evidence?

Yes

Is the work clearly and cogently presented?

Yes

Is the topic of the essay discussed accurately in the context of the current literature?

Yes

Does the essay contribute to the cultural, historical, social understanding of the field?

Yes

Reviewer Expertise:

Cancer nursing, qualitative methodologies.

I confirm that I have read this submission and believe that I have an appropriate level of expertise to confirm that it is of an acceptable scientific standard, however I have significant reservations, as outlined above.

## References

[1] : Troubling gatekeepers: methodological considerations for social research. *International Journal of Social Research Methodology* .2013;16(6) : 10.1080/13645579.2013.823281 457-462 10.1080/13645579.2013.823281

[2] : A screening tool for predicting gatekeeping behaviour. *Nurs Open* .4(4) : 10.1002/nop2.83 187-199 10.1002/nop2.83 29085645PMC5653390

[3] : Negotiating with Gatekeepers: Reflexivity in Exploring Turkish Men’s Engagement in Domestic Violence Perpetrator Interventions in the UK. *The Qualitative Report* .2020; 10.46743/2160-3715/2020.4400 10.46743/2160-3715/2020.4400

# Response

**Published:** 2023-10-24

**Authors:** Marjan Mohammadzadeh

- **The autho** **rs should reframe the discussion on the challenges of the first two approaches (major government and private organisations) in the context of gatekeepers. The discussion could be stren** **gthened by reference to insights provided by Crowhurst and Kenney-MacFoy (2013) and the findings of Snowden and Young (2017) on gatekeeping as a continuum from unconscious to conscious. Some attention to research reflexivity would also strengthen the discussion on accessing participants via gatekeepers and any possible power dynamics. The paper by Turhan and Bernard (2020) offers guidance on approaching reflexive accounts.**

Additional clarifications and explanations concerning the points you requested have been included in the third paragraph of the discussion section.  

- **Rephrasing of the following sentences is needed where the meaning is not entirely clear: a) The last sentence in the section ‘ethical considerations’; ‘ …according to the informed consent forms…’ ; b) Discussion, paragraph 5, the first sentence; what does ‘more and less predictable’ mean?**

The requested modifications have been implemented.  

- **Some minor typos noted as follows: a) Introduction, paragraph 3, sentence beginning with ‘In other words…in one or more characteristic [sic]’; b) Introduction, paragraph 3, sentence beginning with ‘However, scientific studies …instead of the whole populations [sic]’**

The requested modifications have been implemented.
